# Experimental and Analytical Investigations on Tribological Properties of PTFE/AP Composites

**DOI:** 10.3390/polym13244295

**Published:** 2021-12-08

**Authors:** Hai Wang, Annan Sun, Xiaowen Qi, Yu Dong, Bingli Fan

**Affiliations:** 1 School of Mechanical Engineering, Yanshan University, Qinhuangdao 066004, China; wh_manuscript@163.com (H.W.); m15091777129@163.com (A.S.); Fanbingli@ysu.edu.cn (B.F.); 2 Aviation Key Laboratory of Science and Technology on Generic Technology of Self-Lubricating Spherical Plain Bearing, Yanshan University, Qinhuangdao 066004, China; 3 School of Civil and Mechanical Engineering, Curtin University, Perth, WA 6845, Australia; Y.Dong@curtin.edu.au

**Keywords:** polytetrafluoroethylene (PTFE), poly(para-phenyleneterephthalamide) PPTA pulp, tribological properties, transfer films

## Abstract

The tribological properties of polytetrafluoroethylene (PTFE)/AP (poly(para-phenyleneterephthalamide) (PPTA) pulp) composites under different test conditions (load: 2N, 10N; frequency: 1 Hz, 4 Hz; amplitude: 2 mm, 8 mm) were holistically evaluated. PTFE/AP composites with different AP mass ratios of 3%, 6%, and 12% as a skeleton support material were prepared. The coefficient of friction (COF) and wear rate were determined on a ball-on-disk tribometer. Furthermore, the morphology, element composition, and chemical structure of the transfer membrane were analyzed accordingly. The relationships between load, frequency, amplitude, and tribological properties were further investigated. According to the wear mechanism, AP enables effective improvement in the stiffness and wear resistance, which is also conducive to the formation of transfer films.

## 1. Introduction

Polytetrafluoroethylene (PTFE) composites are widely used as lubricating materials on account of good mechanical and tribological properties [1]. However, the unique structure of PTFE results in poor wear resistance, which can be overcome by adding fillers to improve tribological properties by means of nanoparticle filling [2,3], fiber material blending [4,5], and surface modification [6]. Among them, fiber reinforcement has demonstrated rapid growth and development. This is because the matrix can effectively transfer the load to the fibers to improve the bearing capacity of polymer-based composites [7]. Due to the high bond energy of main molecular chains of fibers, greater hardness of fiber surfaces, good thermal properties [8], and high tensile strength [9], the wear resistance can be improved accordingly. Therefore, many types of fibers are widely used in composite materials [10], such as poly-(para-phenylene-2,6-benzobisoxazole) (PBO), poly(para-phenyleneterephthalamide) (PPTA), co-poly-(para-phenylene-3,4′-oxydiphenylene terephthalamide) (PPODTA), and fibers from biodegradable polymers such as poly(lactic acid) (PLA). The reinforcing fibers of PTFE composites include glass fibers (GFs) [11], carbon fibers (CFs) [12], nylon fibers [13], ultra-high-molecular-weight polyethylene (UHMWPE) fibers [14], etc. The type of fiber greatly affects the wear rate of resulting composite materials, especially for PTFE composites. GF can effectively promote the formation of PTFE transfer film and reduce the friction coefficient, while the transfer film can slow down the scraping effect of fiber. CF can improve the binding force between fiber and PTFE matrix and thus improve wear resistance [15]. Compared with CF, Wang et al. further proved that a large amount of interfacial free space along and around basalt fibers resulted in poor bonding strength with PTFE matrix [16]. Jasbir et al. [17] has proved that scraping of GFs does not destroy PTFE-based transfer films. Furthermore, due to abrasive and adhesive wear, the worn surfaces are even self-repaired. As a result, GF can effectively improve the tribological properties of PTFE-based polymers. CF has the strong ability to reduce the true contact areas in friction so that the plough and the adhesion would be reduced inside the friction interfaces [18]. However, the matrices far away from CFs have a dissimilar wear mechanism. The low content of CFs cannot support the load sufficiently. Similarly, UHMWPE fibers can bear most of the load and reduce the shear evolution on the adhesive point during the friction process [19]. Further studies have drawn the conclusion that UHMWPE fibers can be bonded well to the matrices as well as to be wrapped by PTFE, thus leading to the great enhancement of tribological properties of resulting composite materials. The fiber can also reduce the size of PTFE debris and improve the tribological properties of PTFE composites [20].

In recent years, various kinds of aramid fiber derivatives have been developed rapidly, mainly including aramid particles and aramid fiber. Xian et al. [21] reported that aramid particles did not show potential influence on tribology properties. The abrasive wear caused by aramid particles is the deterioration of the friction interface. However, aramid fibers become very popular in the research field of fiber reinforcements [22]. Aromatic fibers are spun from aromatic polyimide resins whose main chains consist of aromatic rings and amide bonds [23]. Aramid fiber can be used as fabric material as reinforcement phase or directly through blending to form high-performance materials. Fabric materials containing aramid fibers have specific strength and excellent tribological properties [24], especially PTFE/aramid fabric composites [25]. Different from aramid particle-reinforced materials, the main wear form of PTFE/aramid fabric composites is adhesive wear, and the more continuous and uniform transfer film can effectively reduce the wear degree [26]. The varieties of aramide fibers which are blended directly as a filler contain poly (p-benzoyl p-phenylenediamine) fiber (PPTA) and poly(p-benzoamide) fiber (PBA) developed by DuPont de Nemours, Inc. (Wilmington, DE, USA) [27], Technora aparetic aramide copolymer fibers (Technora) obtained from Teijin Limited Company (Japan) [28], and aramid fiber 1414 (Shuobang New Performance Materials Technology Co., Ltd., Jiangxi, China) [29]. Among them, PPTA is the most commonly used in composite materials. The skeleton atoms within the molecules are bound by strong covalent bonds, and the hydrogen on the polar intermolecular amide groups can be combined with electron-available carbonyl groups in the amide group on the other chain segment to generate hydrogen bonds, resulting in a high degree of crystallinity. On the other hand, benzene rings form large conjugated bonds that are difficult to rotate, so large molecular chains have linearly rigid extended chains in order to yield high strength and modulus. PPTA pulp is a derivative of PPTA, which maintains high performance and is easier to disperse in liquid phase [30]. Therefore, it is a potential wear-resistant phase material.

In the present work, PTFE/AP composites with different AP mass ratios (i.e., 3%, 6%, and 12%) were prepared and their tribological properties were determined by ball-on-disk tests in relation to the effect of load, frequency, and amplitude. The morphological structures of worn surfaces and transfer films were observed by scanning electron microscopy along with the analysis of elemental content and distribution by means of energy dispersive spectroscopy (EDS). Finally, the wear mechanism suggested that AP plays a very good bearing role as a skeleton, and divides the contact surface into several areas to effectively slow down the wear of PTFE. At the same time, the silane coupling agent solves the problem of poor adhesion between the fiber and the matrix.

## 2. Materials and Methods

### 2.1. Materials

**Figure 1:**
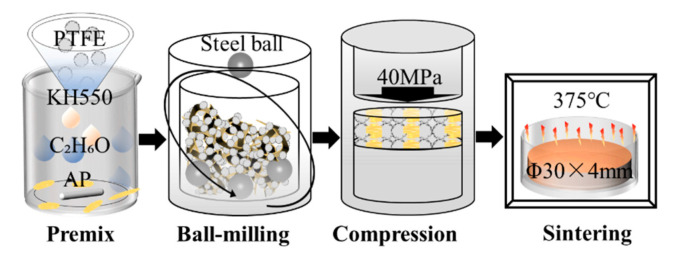
Preparation process of PTFE/AP composites.

AP was provided by Tech-in Materials Co., Ltd., (Nanjing, China). The average length of AP was 1 mm. PTFE with the diameter of 35 μm was supplied from Shanghai 3F New Materials Co., Ltd., (Shanghai, China). Silane coupling agent KH550 was provided by Green Plastic Products Co., Ltd., (Dongguan, China). The detailed preparation process of PTFE/AP composites is illustrated in Figure 1.

AP modified by KH550 and anhydrous ethanol as the solvent were mixed by magnetic stirrer at 300 rpm. Then, PTFE powders were added into the mixture that underwent additional 4 h stirring subsequently. In order to improve the uniformity of PA dispersion and better combination with PTFE, the obtained pretreated material was subjected to secondary ball milling by a ball mill (SPEX 8000D, USA). The extracted powders were placed in a vacuum oven at 100 °C for 12 h. Then, the dried powders were pressed under 3 metric tonnes hydraulic press to turn into disk samples (Φ30 mm × 4 mm). Such samples were sintered at 375 °C and remained warm for 2 h. Finally, there was natural cooling to room temperature in a muffle furnace (SX2-5-12LTP, Lichen, China). The weights of samples with different AP ratios were 6.11 g (3% AP), 5.95 g (6% AP), and 5.74 g (12% AP), respectively. Many studies have shown that the properties of composites begin to decline when the mass ratio of fiber-reinforced phase is greater than 10% [21,24]. Zhang et al. prepared PTFE composites filled with PPTA mass ratio of 1–9% [31]. Higher AP mass content may induce the material/filler agglomeration in the synthesis process resulting in poor homogeneity of composite materials. The surface energy of PTFE is small, which makes it difficult to adsorb and agglomerate. The low content of AP is more conducive to mutual dispersion, which promotes the formation of cross-linking network structure.

### 2.2. Determination of Tribological Properties

The tribological performance was evaluated by a ball-on-disk tribometer (CSM, Austria) at room temperature in a test mode based on the reciprocating movement. The upper specimen was YG6 ball (i.e., 94% tungsten carbide and 6% carbonic oxide) with a ball diameter of 6 mm. Prior to the tests, the specimens were polished using 1000#, 2000# abrasive papers and ultrasonically cleaned with the absolute alcohol for 15 min. Each test was repeated at least three times to warrant the test reproducibility with reported average data and associated standard deviations. Twelve groups of tests were designed according to different test conditions according to Table 1.

A multifunctional precision coating material performance tester (Anton Paar, Austria) was employed to characterize the worn surface morphology of PTFE/AP composites, and proprietary software (Image Plus) was utilized to calculate the cross-sectional area of the composites. The cross-sectional areas on five different positions were selected to calculate the average dimensions. The calculation formula is given as follows:

ΔV = Sd(1)

W = ΔV/(F × L)(2)

where W is the wear rate (m^3^/N·m), S is the mean cross-sectional area of wear tracks (μm^2^), d is the length of wear tracks(mm), F is the test load (N), and L is the sliding distance (m).

**Figure 2:**
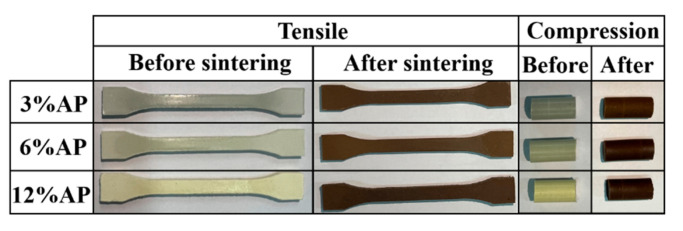
Photos of tensile (and compression) specimen before (and after) sintering.

A scanning electron microscope (SEM, Phenom Nano, The Netherlands) using energy dispersive X-ray spectroscopy (EDS) was implemented to perform the morphologies of worn surfaces and the element distribution of the transfer films. The density of composite materials was determined by an electronic density detector (BSA2245, Sartorius, Germany). The hardness of the composites was measured using a Shore D hardness tester (HSD, Hitech (Qingdao) Enterprise Inc., Qingdao, China). The mechanical properties of the PTFE/AP composites were tested using a microcomputer-controlled electronic universal testing machine (WDW-100E, Jinan Hensgrand Instrument Co., Ltd., Jinan, China) at a loading speed of 2.0 mm/min. Photos of specimens required for the test are shown in Figure 2. The key input parameters of the tensile test specimens are 5 mm in width and 4 mm in thickness and the size of the compressed sample is Φ8 × 15 mm.

The chemical structures of transfer films were tested by a Fourier transform infrared spectrometer (FTR-IR, iS5, Thermo Scientific Nicolet, Waltham, MA, USA). The average value of each group performance test was taken five times.

## 3. Results

### 3.1. Mechanical Properties and Physical Properties

**Figure 3:**
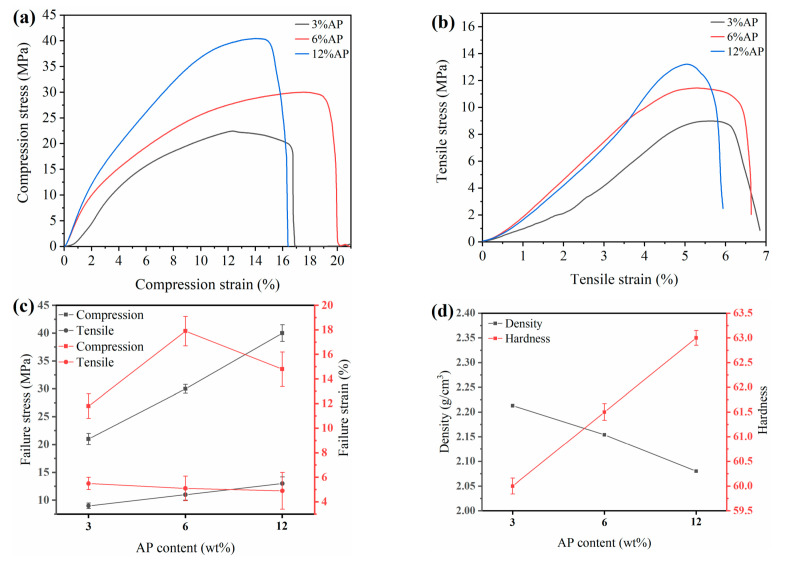
The friction coefficient of (**a**) compression stress–strain curves, (**b**) tensile stress–strain curves, (**c**) curves of failure stress and failure strain, and (**d**) density and hardness.

Figure 3a,b show the compression and tensile stress–strain curves of PTFE/AP composites, respectively. With the increase in AP content, the tensile and compressive properties of composites are improved, which undoubtedly plays a positive role in tribological properties. The compressive strength increases by 10 MPa and the tensile strength increases by 2 MPa with the increase in AP content by two times. Failure stress and failure strain were obtained from the stress–strain curves shown in Figure 3c. The increase in AP content results in the decrease in tensile failure strain. This is because the decrease in density (Figure 3d) and crosslinking degree leads to a decrease in toughness and increase in brittleness. With the increase in AP content, the non-uniform dispersion of AP in the PI matrix causes structural defects, leading to stress accumulation. When the AP content reaches 12%, the adhesion between it and the substrate is severely weakened, and the decrease in bonding strength leads to a decrease in the overall performance of the material. The densities were determined to be 2.213, 2.154, and 2.080 g/cm^3^ at the AP mass ratios of 3%, 6%, and 12%, respectively. The surface hardness of PTFE/AP composites increases by 1.5 HD every time the AP content increases by two time. The increase in hardness enhances the bearing capacity and wear resistance effectively.

### 3.2. Friction and Wear

**Figure 4:**
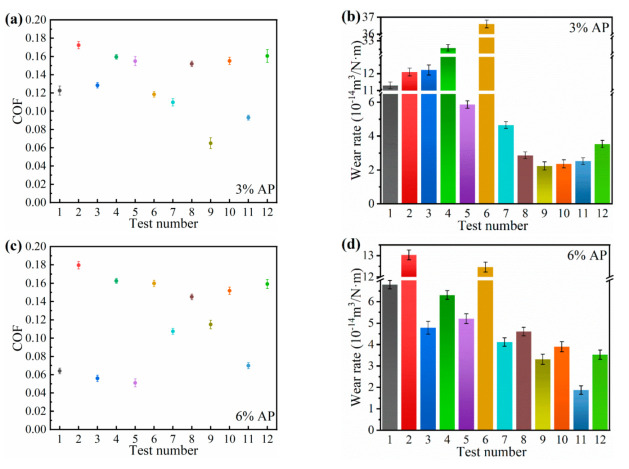

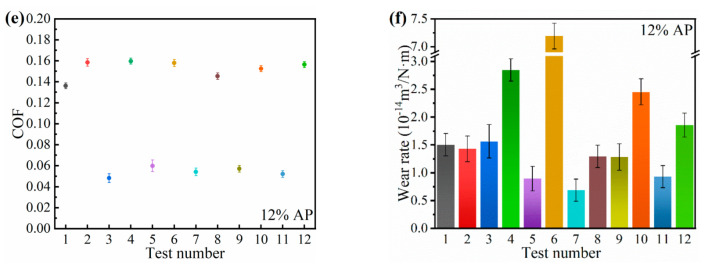
The friction coefficient of (**a**) 3% AP, (**c**) 6% AP, (**e**) 12% AP and wear rate of (**b**) 3% AP, (**d**) 6% AP, (**f**) 12%AP.

The coefficient of friction and wear rate of PTFE/AP composites are shown in Figure 4. The most intuitive feature is that the COFs are enhanced with increasing the test frequency (Figure 4a). Moreover, by increasing AP mass ratio, such a reduction trend becomes more evident (Figure 4c,e). The increase in test frequency accelerates the destruction of composite surfaces and further increases the increment of friction temperature, thus resulting in phenomenal wear intensification. COFs reveal a decreasing tendency with the load reduction at the amplitude of 8 mm. Conversely, decreasing the load leads to an increase in COF at the amplitude of 2 mm. A smaller amplitude arises from shorter slip distance of steel ball per unit time to generate a stick-slip vibration state for PTFE/AP composites that are deemed as an elastic solid. Both smaller load and amplitude result in the easy formation of transfer films on the steel ball surfaces leading to increasing the COFs.

Figure 4b–f indicate that wear rate is inversely proportional to AP content. When AP content increases twice, wear rate decreases the same accordingly. The wear rate at the amplitude of 2 mm is lower than that at 8 mm. In particular, the wear rate of PTFE/AP composites with the inclusion of 3% AP at the amplitude of 2 mm can be about twice as low as that at 8 mm, as illustrated in Figure 4b. At short amplitudes and low loads, the high frequency results in high wear rates and the pattern is not significant. For example, the wear rate of 10# (2.3639 × 10^−14^ m^3^/N·m) is higher than 9# (2.243 × 10^−14^ m^3^/N·m). As well as the load, the wear rate of 8# (2.8637 × 10^−14^ m^3^/N·m) is higher than 11# (2.5182 × 10^−14^ m^3^/N·m). However, this trend becomes relatively slow with increasing the AP content. When taken as the reinforcing materials, AP enables effective promotion of the hardness and stiffness of PTFE/AP composites while reducing plasticity for better wear resistance. It is manifested that there is a proportional relationship between the frequency and wear rate. For instance, the wear rate of PTFE/AP composites with the inclusion of 12% AP at the frequency of 4 Hz is almost twice as high as the counterpart at 1 Hz. A high frequency inevitably induces an increase in the number of scratches of the same length, which further aggravates the damage to PTFE/AP composites. As such, friction temperature is increased and oxidation reaction becomes intensified, leading to the difficult formation of high-quality transfer films.

### 3.3. Wear Morphology

**Figure 5:**
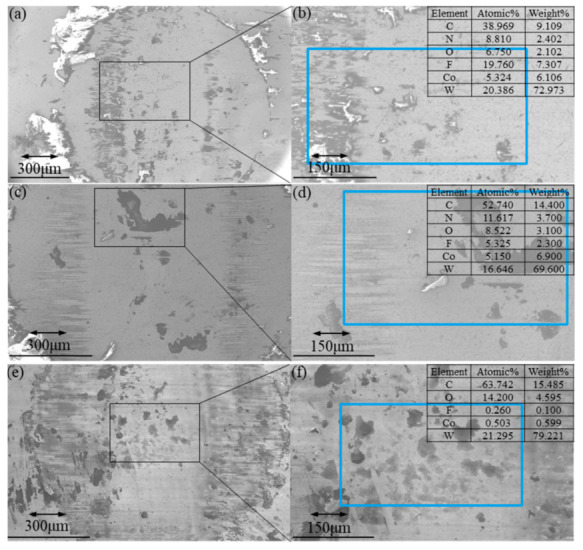

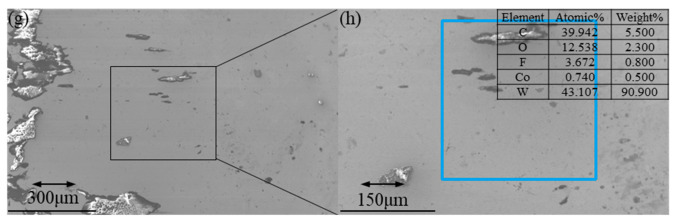
Worn surface of YG6 ball against 3% AP composites (**a**,**b**): At 7# condition, (**c**,**d**) at 8# condition, (**e**,**f**) pure PTFE at 7# condition, (**g**,**h**) pure PTFE at 8# condition.

Figure 5 demonstrates the counter surface morphology of PTFE/3% AP composites along with main-element distribution, where Figure 5a–d refer to test numbers 7 and 8, respectively. Figure 5e,f are the control group (unfilled PTFE) of Figure 5a,b, and 5g,h are the control group (unfilled PTFE) of Figure 5c,d.

The COFs of 7# and 8# conditions sliding against 3% AP/PTFE composites are 0.1098 and 0.1519, respectively. When increasing the frequency from 1 to 4 Hz, the COF would be increased by 38.34%. When the surface contains more fluorine, the transfer films cover a larger and more continuous area, resulting in a lower friction coefficient [32]. The atomic and weight concentration of fluorine on the worn surface are 19.760% and 7.307% for 7# (0.260% and 0.100% for pure PTFE), and 5.325% and 2.300% for 8# (3.672% and 0.800% for pure PTFE). Combined with Figure 4, the high frequency prevents the formation of transfer films from causing high COF. The contents of carbon and oxygen have proven that high frequency also enables to strengthen the oxidation reaction of friction interfaces. However, there is almost no fluorine on the worn surface against pure PTFE, which verifies that the low surface energy of pure PTFE is difficult to form a transfer to the dual surface, indicating that PTFE/AP is beneficial to the formation of transfer film.

**Figure 6:**
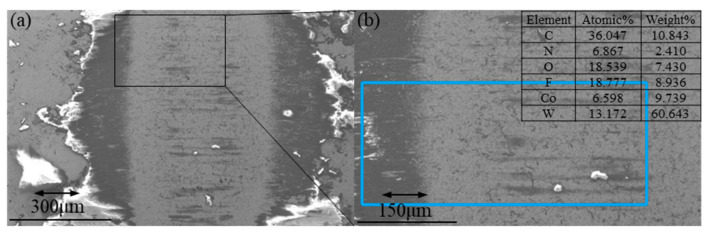

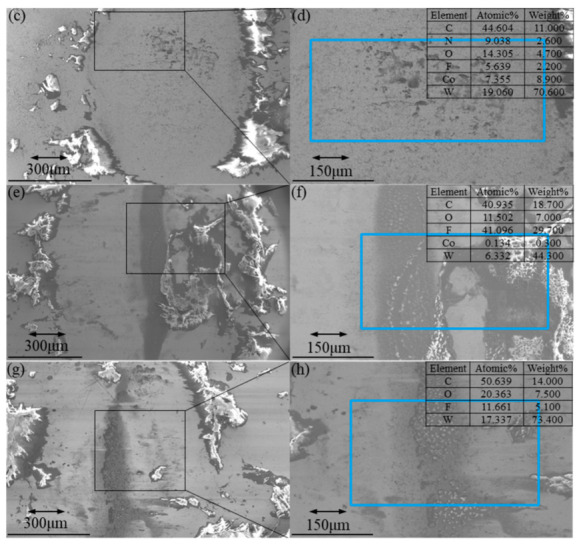
Worn surfaces of YG6 ball against 12% AP composites (**a**,**b**): At 4# condition, (**c**,**d**) at 6# condition, (**e**,**f**) pure PTFE at 5 at 7# condition, (**g**,**h**) pure PTFE at 6# condition.

The friction coefficient determined via deformation energy analysis is in good accordance with a classical tribological theory that the friction force is proportional to the applied load. When the normal load increases, PTFE/AP composites yield higher contact stress and plastic deformation while the sliding process requires more energy. Figure 6a–d reveal the worn surfaces of composites with the inclusion of 12% AP at the same frequency of 4 Hz under two different loads of 5 and 2N, respectively. A large amount of PTFE/AP composite debris has been observed to be accumulated at the edge of worn surfaces, which is attributed to the enrichment of fluorine. Due to the poor distribution of fluorine in the central region, as well as a high proportion of tungsten, a very thin and discontinuous transfer film is formed at the friction interface despite the existence of major bare steel surfaces. The formation of transfer films is hindered by more carbon ratios, suggesting the difficult aggregation of fluorine at its low concentration. Figure 6e–h show that the long amplitude is favorable for the formation of transfer film. The interfacial friction temperature of pure PTFE samples rises higher, and the increase in carbon and fluorine contents proves that the interfacial reaction intensifies.

In comparison (Table 2), it has been found that both friction coefficient and wear rate of PPTA/AP composites obtained in this study are similar or lower than those according to previous investigations [13,19,33].

### 3.4. Wear Mechanism

**Figure 7:**
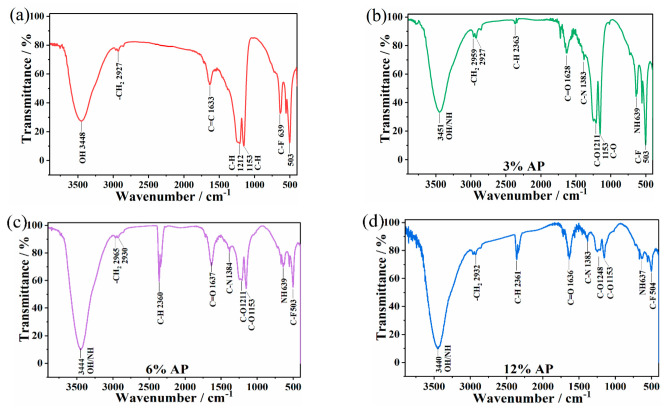
FTR-IR of transfer films (**a**) Pure PTFE, (**b**) 3%AP, (**c**) 6%AP and (**d**) 12%AP.

The chemical structure of the transfer film based on PTFE/AP composites at different AP ratios was determined by FTR-IR spectra in Figure 7. The strong absorption peak at 3448 cm^−1^ is assigned to the stretching vibration peak of O-H bond, which is caused by the adsorption during the formation of transfer films. The absorption peak at 2927 cm^−1^ represents the stretching vibration peak of saturated C-H bond. C=C stretching vibration peak at 1633 cm^−1^, C-H asymmetric stretching vibration peak at 1212 cm^−1^ and 1153 cm^−1^, and C-F bond at 639 cm^−1^ and 503 cm^−1^ are typical PTFE peaks [33]. The stretching vibration peak of amide A band from the N-H bond is represented at 3340 cm^−1^ in Figure 7b. Amide III band at 1636 cm^−1^ is mainly induced by C=O stretching vibration. The associated peak at 1383 cm^−1^ is referred to the amide II band, which is mainly ascribed to the coupling of C-N stretching vibration and N-H bending vibration. The peak at 1248 cm^−1^ denotes the amide I band owing to the superimposed peaks of CH_2_ rocking vibration, N-H bending vibration, and C-N stretching vibration. These peaks indicate the presence of amide groups in transfer films. The absorption peak at 2932 cm^−1^ is associated with the stretching vibration peak of saturated C-H bond [17]. The weak absorption peak at 2361 cm^−1^ can arise from the overtone of C-H stretching vibration. The peak location is close to Figure 7b in Figure 7c,d despite different relative peak intensities. The O-H/N-H bond strength near 3440 cm^−1^ appears to be the highest (Figure 7d), indicating the occurrence of more N-H, which is consistent with AP mass ratio. The C-H bond at 2360 cm^−1^ has achieved the highest strength as opposed to a low strength in relation to C-F bond at 503 cm^−1^, which infers the significant deformation of C-H bond. It is proven that silane coupling agent is firstly combined with PPTA-AP as the bonded framework material to generate a much stronger chemical bond at the interface for the improvement of bonding strength. Meanwhile, such a couple agent is also beneficial for effectively enhancing the dispersibility of the solvent along with the reduction in agglomeration in the preparation process of PTFE/AP composites.

The symmetrical helical conformation of PTFE results in poor mechanical properties and creep resistance. However, the rigidity of helical conformation chains is very high with a bending difficulty. Meanwhile, the high crystallinity of PTFE leads to flaky spalling and poor wear resistance in dry friction.

**Figure 8:**
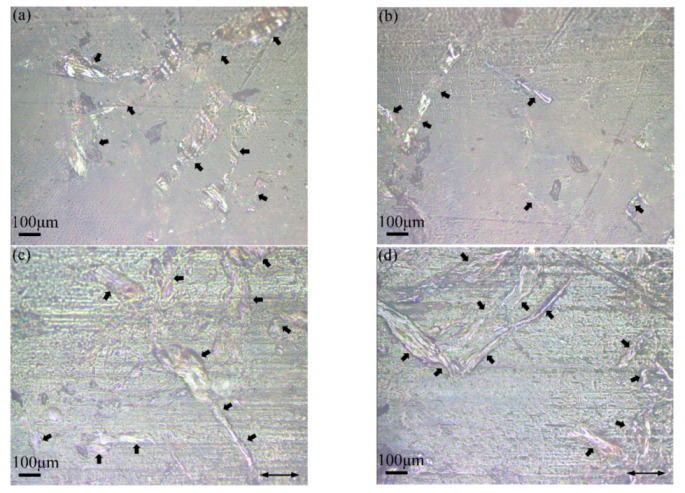
Images of PTFE/AP composites: (**a**,**b**) outside and (**c**,**d**) inside the wear track.

It can be seen from Figure 8a,b that AP has been steadily embedded in PTFE matrices, resulting in the improvement of mechanical properties (Figure 8c,d) and wear resistance. The arrows in the Figure 6 point to the AP on the wear track of PTFE/AP composites. The AP inside the wear track is divided into smaller sizes under the combined action of friction, pression, and shear force, which further improves the stiffness and wear resistance of PTFE/AP composites. Furthermore, AP also promotes the speed and intensity of chemical reaction at the friction interface, making the formation of transfer film easier and thus reducing the COF.

**Figure 9:**
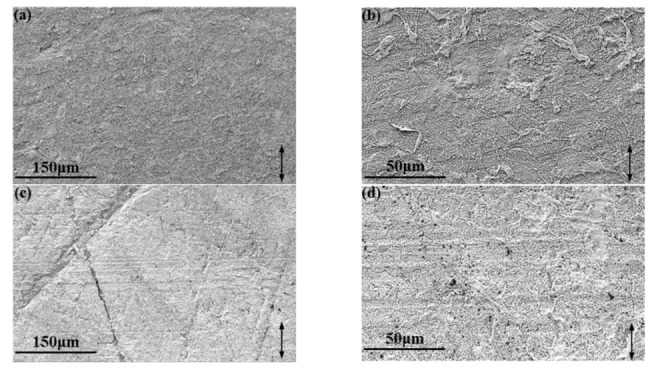
Worn surfaces of PTFE/AP composites: (**a**,**b**) 3% AP at 8# condition and (**c**,**d**) 12% AP at 6# condition.

Figure 9 demonstrates the wear-track morphologies of PTFE/AP composites. As a material skeleton, AP has been well dispersed in PTFE matrices. With increasing the AP content, the ratio of skeleton becomes much larger, and KH550 strengthens the bonding between the skeleton and matrices in order to enhance the mechanical properties of composites. In contrast, larger loads make it easier for AP on the worn surfaces to escape from the substrate (Figure 9a,b) leading to curl deformation. Local sliding friction can be changed to rolling friction for purpose of the reduction in friction coefficient. On the other hand, exposed AP and worn PTFE debris are more likely to form AP–PTFE lubrication film using shorter stroke under the physical action, which can effectively avoid adhesion wear and fatigue wear in order to diminish the wear rate. The apparent grooves illustrated in Figure 9c are indicative of adhesion wear of polymers. The high AP ratio results in much fewer PTFE fragments separated by the wear of the matrices. Accordingly, this prevents the stroke of AP–PTFE lubricating films while the smaller load also reduces the equivalent rolling friction effect of AP. In addition, longer reciprocating distance also slows down the transfer of PTFE to the counterface. The rough counterface further aggravates the wear of PTFE/AP composites. Cracks are a typical characteristic of fatigue wear identified in polymers, such cracks originate from holes and pitting corrosion. The skeleton effect of AP can effectively slow down the crack formation of wear surface and weaken the fatigue wear. Such combined effects contribute to the highest wear rate determined in sample 6# for PTFE/12% AP composites.

## 4. Conclusions

In conclusion, PTFE/AP composites with different AP mass ratios were successfully prepared along with the determination of their tribological properties. The COFs are comparatively decreased with increase in the load at 8 mm (amplitude) and decrease in the load at 2 mm (amplitude). Apparently, both smaller load and amplitude benefit the easy formation of transfer films. It is manifested that increasing the AP mass ratio decreases the wear rate that is proportional to the frequency. A high frequency yields the increase in the number of scratches of the same length, resulting in the aggravated damage taking place in PTFE/AP composites. According to morphological and elemental analyses, oxidation reaction is intensified and the formation of transfer films becomes very difficult at a high frequency. The formation of discontinuous transfer films is believed to be associated with the poor distribution of fluorine in the central region of worn counter-surfaces. As the difficult aggregation of fluorine at its low concentration, the formation of transfer films can be inhibited by the additional carbon. Furthermore, in view of FTR-IR spectra, silane coupling agent plays an important part in improving the bonding of AP as the skeleton and PTFE matrices. Overall, AP can be confirmed to be vital reinforcing materials to increase both stiffness and wear resistance of composite materials, which is directly conducive to the formation of transfer films. PTFE/AP composites has the advantages of simple preparation, lightweight, good lubricity, and low wear rate. It can be used as a self-lubricating material in various tribocomponents, such as self-lubricating bearings.

## Figures and Tables

**Table 1:** Tribology test number.

Test Number	Load (N)	Frequency (Hz)	Amplitude (mm)
1#	10	1	8
2#	10	4	8
3#	5	1	8
4#	5	4	8
5#	2	1	8
6#	2	4	8
7#	10	1	2
8#	10	4	2
9#	5	1	2
10#	5	4	2
11#	2	1	2
12#	2	4	2

**Table 2:** Comparison of references.

	Tensile Strength (Mpa)	COF	Wear Rate (10^−14^ m^3^/N·m)
PTFE + 20%glass fiber [7]	19.3	0.19	1.1
PTFE + 20% Carbon fibers (D = 7 μm) [13]	18.99	0.418	0.021
PTFE + 20% Basalt fibers (D = 9 μm) [13]	15.68	0.211	0.124
PTFE + 20% Serpentine + 10% UHMWPE fibers [18]		0.316	0.12
PTFE + 3% Aramid fibers (D = 12 μm) [19]		0.703	0.82
PTFE + 2% Carbon fibers (D = 7 μm) [33]	18.74	0.174	60.4
PTFE + 2% Carbon fibers (D= 200 nm) [33]	21.87	0.151	58.2
PTFE + 2% Multiwalled carbon nanotube [33]	11.19	0.159	18.3
PTFE + 15% Potassium titanate whiskers [34]	23.7	0.15	0.12
PTFE + 15% Carbon fibers (D = 9 μm) [34]	20.18	0.121	0.23

## Data Availability

Data are contained within the article.
